# Prognostic nomogram for bladder cancer with brain metastases: a National Cancer Database analysis

**DOI:** 10.1186/s12967-019-2109-7

**Published:** 2019-12-09

**Authors:** Zhixian Yao, Zhong Zheng, Wu Ke, Renjie Wang, Xingyu Mu, Feng Sun, Xiang Wang, Shivank Garg, Wenyin Shi, Yinyan He, Zhihong Liu

**Affiliations:** 1 Department of Urology, Shanghai General Hospital, Shanghai Jiao Tong University School of Medicine, 100 Haining Road, Shanghai, 200080 People’s Republic of China; 2 Department of Radiation Oncology, Sidney Kimmel Medical College at Thomas Jefferson University, Sidney Kimmel Cancer Center, Philadelphia, PA USA; 3 Department of Obstetrics and Gynecology, Shanghai General Hospital, Shanghai Jiao Tong University School of Medicine, 100 Haining Road, Shanghai, 200080 People’s Republic of China

**Keywords:** Bladder cancer, Brain metastasis, Machine learning, Nomogram, Overall survival

## Abstract

**Background:**

This study aimed to establish and validate a nomogram for predicting brain metastasis in patients with bladder cancer (BCa) and assess various treatment modalities using a primary cohort comprising 234 patients with clinicopathologically-confirmed BCa from 2004 to 2015 in the National Cancer Database.

**Methods:**

Machine learning method and Cox model were used for nomogram construction. For BCa patients with brain metastasis, surgery of the primary site, chemotherapy, radiation therapy, palliative care, brain confinement of metastatic sites, and the Charlson/Deyo Score were predictive features identified for building the nomogram.

**Results:**

For the original 169 patients considered in the model, the areas under the receiver operating characteristic curve (AUC) were 0.823 (95% CI 0.758–0.889, P < 0.001) and 0.854 (95% CI 0.785–0.924, P < 0.001) for 0.5- and 1-year overall survival respectively. In the validation cohort, the nomogram displayed similar AUCs of 0.838 (95% CI 0.738–0.937, P < 0.001) and 0.809 (95% CI 0.680–0.939, P < 0.001), respectively. The high and low risk groups had median survivals of 1.91 and 5.09 months for the training cohort and 1.68 and 8.05 months for the validation set, respectively (both P < 0.0001).

**Conclusions:**

Our prognostic nomogram provides a useful tool for overall survival prediction as well as assessing the risk and optimal treatment for BCa patients with brain metastasis.

## Background

As the top ranked malignancy of the urinary system, bladder cancer (BCa) incidence data in the US shows an estimated 79,030 (8th among all sites) new cases and 16,870 (8th among all sites) deaths in 2017 [1]. Unfortunately, 10–15% of BCa patients already have metastasis at initial diagnosis and 15–30% high-grade BCa will eventually progress to advanced disease and lead to poor prognosis [2].

Despite an initial response to chemotherapy, patients with non-organ-confined disease fail to attain satisfactory survival [3]. Since no optimally effective chemotherapeutic modality has been found, patients with NOC disease can barely survive for more than 3–6 months [4]. According to a previous population-based study of the SEER database, only 4.1% (76/1862) BCa patients had brain metastases in a cohort of 1862 patients with metastatic sites [5]. Given the rarity of brain metastases at presentation, currently, there is no randomized phase II or III clinical trials exploring outcomes of this group. The survival prognosis of this subgroup calls for significant melioration when compared to those with cerebral metastasis from other malignancies [6].

Some reports have claimed that stereotactic radiosurgery and whole-brain irradiation can be a useful alternative approach for patients with brain metastasis in certain malignancies [7, 8]; however, suitable treatment for BCa patients with brain metastases remains unclear. A study conducted in 2002 in Cleveland retrospectively analyzed 16 BCa patients with brain metastases and suggested more aggressive treatment rather than radiation therapy alone [9]. However, the cohort was too small to extract robust clinical traits. In 2010, Fokas et al. found no significant difference in survival after comparing radiotherapy alone with radiotherapy plus surgery in 62 patients with brain metastases from BCa [10]. Therefore, reconsideration of current medical strategies is indispensable, since the role of surgeries of the primary tumor or radiotherapy of brain lesions in the treatment of metastatic BCa is still obscure.

Although previous studies have identified several prognostic factors of poor outcome in advanced BCa, such as the presence of visceral metastasis, anemia, and C-reactive protein (CRP) [3, 11, 12], it remains unknown whether they could be applied to the clinical assessment. Currently, prognostic nomograms are widely applied as prognostic devices in oncologic medicine. With the ability to incorporate clinical characteristics to generate individual probabilities of clinical events, nomograms can aid clinical decisions and facilitate our drive towards personalized medicine [13]. The purpose of our study was to create a nomogram predicting overall survival (OS) of BCa patients with brain metastasis and evaluate suitable therapeutic modalities for this cohort.

## Materials and methods

### Study population

The National Cancer Database (NCDB) was queried for patients initially diagnosed with histological confirmed BCa (topographical code C67, International Classification of Diseases for Oncology, 3rd edition) between 2004 and 2015. Patients with brain metastatic disease at the time of presentation were selected for the analysis.

**Fig. 1:**
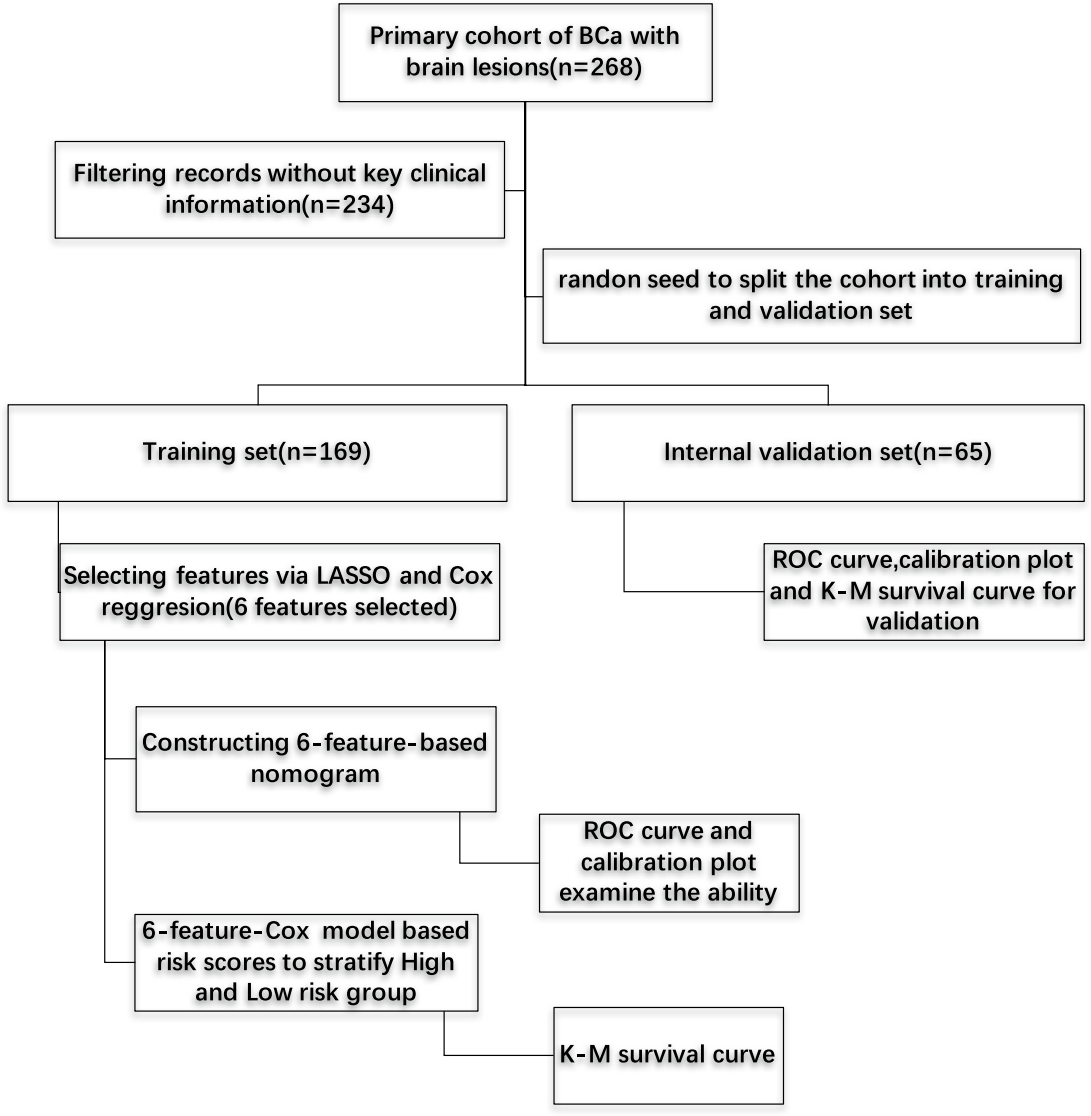
Flowchart of the analysis

Baseline medical traits (including age; sex, race; pathological grade; tumor histology, lymph node vascular invasion, and clinical stage [TNM] of the American Joint Committee on Cancer; surgical statuses of the primary and metastatic sites; chemotherapy; radiation therapy; and palliative care) were derived from medical records (Table 1). Other inclusion criteria were as follows: age > 18 years; BCa as the primary cancer diagnosis; brain metastasis; other distant metastatic sites including bone, liver, lung, and distant lymph node involvement; active follow-up; and patients with > 30 days of survival. Patients without sufficient information about distant metastatic sites or survival data were excluded. No detailed data were available regarding the specific types of chemotherapy or hormonal therapy or palliative care agents. Finally, we included 234 patients with the above-mentioned criteria. We used a computer-generated random seed to assign 169 of these patients to the training set, and 65 patients to the internal testing set for subsequent analysis. Work of flow is displayed in Fig. 1.

**Table 1:** Description of clinical characteristics and their values

Clinical variables	Description	Values
Age	Age of the patient at diagnosis	< 65 years or ≥ 65 years
Sex	The gender of the patient	Male or female
Race	The primary race of the person	White, black or others
Grade	Describes the tumor’s resemblance to normal tissue (coded according to ICD-O-3)	Well differentiated, poorly differentiated or Unknown
Tumor_Stage	NCDB analytic stage identifies the clinically or pathologically determined size and/or extension of the primary tumor (cT) as defined by the American Joint Committee on Cancer (AJCC)	High (Stage III, IV) or low (Stage I, II)
Lymph_nodes	Identifies the clinically-determined absence or presence of regional lymph node metastasis and describes the corresponding extent as defined by the American Joint Committee on Cancer (AJCC)	Yes, no or unknown
Histology	Indicate the pathological histology of tumor cells (coded according to ICD-O-3)	Transitional cell carcinoma, papillary urothelial carcinoma, small cell carcinoma or others
Lymph_Vas_invasion	Indicate the presence or absence of tumor cells in lymphatic channels (other than lymph nodes) or blood vessels within the primary tumor as noted microscopically by the pathologist	Yes, no or unknown
Met_Bone	Indicate the presence of distant involvement of bone at the time of diagnosis	Yes or no
Met_Liver	Indicate the presence of distant involvement of liver at the time of diagnosis	Yes or no
Met_Lung	Indicate the presence of distant involvement of lung at the time of diagnosis	Yes or no
Surgery_Primary	Records the surgical procedure and approach performed to the primary site	Minimal invasive surgery, non-minimal invasive surgery or no surgery
Chemotherapy	Records of chemotherapy administrated as first course treatment	Yes or no
Radiation_Therapy	Anatomic target volume is directed at tumors lying within the substance of brain or its meninges	Yes or no
Paliative_Care	Any care provided an effort to palliate or alleviate symptoms	Yes or no
Brain_Confined_Met	Indicate the presence of distant involvement of brain only or brian combined with other organs at the time of diagnosis	Brain confined or non-brain confined
CDCC_Score	Charlson/Deyo Score, a weighted score derived from the sum of the scores for each of the comorbid conditions listed in the Charlson Comorbidity Score Mapping Table (source http://dx.doi.org/10.17632/nn6y58v8vv.1#file-a72735e9-15b5-4a10-aef5-deddad2463e8)	0–3
Surgery_Met	Records the surgical removal of distant lymph nodes or other tissues or organs beyond the primary site	Yes or no

### Compliance with ethical standards

The NCDB is a hospital-based registry of cases treated at American College of Surgeons Commission on Cancer accredited cancer programs. Extraction of data from the NCDB did not require extra informed consent. All the data were downloaded at the Sidney Kimmel Cancer Center of Thomas Jefferson University.

### Statistical analysis

For feature selection, we used the least absolute shrinkage and selection operator (LASSO) method, which is a machine learning method suitable for the reduction of high-dimensional data [14]. Eighteen variables were used to select the most useful predictive features from the primary data set. The LASSO regression model analysis was performed using the *glmnet* package of R (R Foundation for Statistical Computing, Vienna, Austria).

Univariate and multivariate Cox regression analysis were performed to explore the independent prognostic factors via the *survival* package of R. The Schoenfeld residuals method was applied to test the proportional hazards assumption for the Cox regression model fit. Each regression coefficient of selected variables was converted to a 0 to 100 scale proportion according to its contribution. These points were added across enrolled variables to generate total points, which were then transformed to predicted probabilities. For clinical use, the predictive performance of the nomogram was measured via time-dependent receiver operating characteristic (ROC) analysis with area under the curve (AUC) values. Calibration was employed with bootstrapping to decrease the bias of over-fitting. The *x*-axis represented the prediction calculated using the nomogram, and the *y*-axis the actual risk odds for the individual. The 45-degree line represented an ideal performance of the nomogram, in which the predicted outcome perfectly corresponded with the actual outcome. The model that incorporated the above independent predictors was developed and presented as the nomogram. Nomogram and calibration plots were obtained using the *rms* and *nomogramEx* packages of R.

Survival analysis was performed using the Kaplan–Meier method to probe the correlation between variables and OS, and the log-rank test was performed to compare survival variance in different groups. Decision curve analysis were performed to compare with the current AJCC TNM staging system. All statistical tests and analyses were performed in R software version 3.5.1. Statistical significance was set at < 0.05.

## Results

### Clinicopathologic characteristics

During the study procedure, 268 consecutive BCa patients with brain-involvement were identified from the NCDB. Of these, 234 patients with brain metastasis in accordance with the inclusion criteria were enrolled, and 169 and 65 patients were randomly divided into the training and internal validation cohorts, respectively. The clinicopathologic characteristics and baseline data in the primary and validation cohorts are provided in Table 2. The median follow-up time was 3.38 (range: 1.08–61.21) months.

**Table 2:** Baseline characteristics and distribution of risk stratification of patients in the training and validation cohorts

Characteristics	Training set (%)			Internal testing set (%)		
	Number of cases	High risk	Low risk	Number of cases	High risk	Low risk
Age						
< 65 years	71 (42)	32 (18.9)	39 (23.1)	22 (33.8)	11 (16.9)	11 (16.9)
≥ 65 years	98 (58)	49 (29)	49 (29)	43 (66.2)	21 (32.3)	22 (33.8)
Sex						
Male	128 (75.7)	63 (37.3)	65 (38.5)	47 (72.3)	19 (29.2)	28 (43.1)
Female	41 (24.3)	18 (10.7)	23 (13.6)	18 (27.7)	13 (20)	5 (7.7)
Race						
White	148 (87.6)	70 (41.4)	78 (46.2)	59 (90.8)	29 (44.6)	30 (46.2)
Black	16 (9.5)	8 (4.7)	8 (4.7)	4 (6.2)	3 (4.6)	1 (1.5)
Others	5 (3)	3 (1.8)	2 (1.2)	2 (3.1)	0 (0)	2 (3.1)
Grade						
Well differentiated	14 (8.3)	10 (5.9)	4 (2.4)	1 (1.5)	0 (0)	1 (1.5)
Poorly differentiated	96 (56.8)	39 (23.1)	57 (33.7)	39 (60)	19 (29.2)	20 (30.8)
Unknown	59 (34.9)	32 (18.9)	27 (16)	25 (38.5)	13 (20)	12 (18.5)
Histology						
TCC	94 (55.6)	49 (29)	45 (26.6)	38 (58.5)	17 (26.2)	21 (32.3)
PUC	42 (24.9)	15 (8.9)	27 (16)	8 (12.3)	5 (7.7)	3 (4.6)
SCC	10 (5.9)	3 (1.8)	7 (4.1)	7 (10.8)	2 (3.1)	5 (7.7)
Others	23 (13.6)	14 (8.3)	9 (5.3)	12 (18.5)	8 (12.3)	4 (6.2)
Tumor_Stage						
Low	13 (7.7)	7 (4.1)	6 (3.6)	4 (6.2)	2 (3.1)	2 (3.1)
High	156 (92.3)	74 (43.8)	82 (48.5)	61 (93.8)	30 (46.2)	31 (47.7)
Lymph_nodes						
No	87 (51.5)	39 (23.1)	48 (28.4)	33 (50.8)	16 (24.6)	17 (26.2)
Yes	36 (21.3)	14 (8.3)	22 (13)	17 (26.2)	9 (13.8)	8 (12.3)
Unknown	46 (27.2)	28 (16.6)	18 (10.7)	15 (23.1)	7 (10.8)	8 (12.3)
Lymph_Vas_Invasion						
No	31 (18.3)	12 (7.1)	19 (11.2)	12 (18.5)	5 (7.7)	7 (10.8)
Yes	29 (17.2)	13 (7.7)	16 (9.5)	6 (9.2)	3 (4.6)	3 (4.6)
Unknown	109 (64.5)	56 (33.1)	53 (31.4)	47 (72.3)	24 (36.9)	23 (35.4)
Met_Bone						
No	112 (66.3)	47 (27.8)	65 (38.5)	44 (67.7)	26 (40)	18 (27.7)
Yes	57 (33.7)	34 (20.1)	23 (13.6)	21 (32.3)	6 (9.2)	15 (23.1)
Met_Liver						
No	129 (76.3)	61 (36.1)	68 (40.2)	50 (76.9)	25 (38.5)	25 (38.5)
Yes	40 (23.7)	20 (11.8)	20 (11.8)	15 (23.1)	7 (10.8)	8 (12.3)
Met_Lung						
No	100 (59.2)	40 (23.7)	60 (35.5)	34 (52.3)	16 (24.6)	18 (27.7)
Yes	69 (40.8)	41 (24.3)	28 (16.6)	31 (47.7)	16 (24.6)	15 (23.1)
Surgery_Primary						
Minimal invasive	65 (38.5)	27 (16)	38 (22.5)	31 (47.7)	19 (29.2)	12 (18.5)
No	83 (49.1)	48 (28.4)	35 (20.7)	29 (44.6)	9 (13.8)	20 (30.8)
Non-minimal invasive	21 (12.4)	6 (3.6)	15 (8.9)	5 (7.7)	4 (6.2)	1 (1.5)
Chemotherapy						
No	97 (57.4)	77 (45.6)	20 (11.8)	36 (55.4)	28 (43.1)	8 (12.3)
Yes	72 (42.6)	4 (2.4)	68 (40.2)	29 (44.6)	4 (6.2)	25 (38.5)
Radiation_Therapy						
No	89 (52.7)	50 (29.6)	39 (23.1)	38 (58.5)	14 (21.5)	24 (36.9)
Yes	80 (47.3)	31 (18.3)	49 (29)	27 (41.5)	18 (27.7)	9 (13.8)
Palliative_Care						
No	122 (72.2)	62 (36.7)	60 (35.5)	47 (72.3)	21 (32.3)	26 (40)
Yes	47 (27.8)	19 (11.2)	28 (16.6)	18 (27.7)	11 (16.9)	7 (10.8)
Brain_Confined_Met						
No	67 (39.6)	24 (14.2)	43 (25.4)	22 (33.8)	13 (20)	9 (13.8)
Yes	102 (60.4)	57 (33.7)	45 (26.6)	43 (66.2)	19 (29.2)	24 (36.9)
CDCC_Score						
0	116 (68.6)	50 (29.6)	66 (39.1)	46 (70.8)	23 (35.4)	23 (35.4)
1	37 (21.9)	19 (11.2)	18 (10.7)	12 (18.5)	8 (12.3)	4 (6.2)
2	10 (5.9)	7 (4.1)	3 (1.8)	5 (7.7)	1 (1.5)	4 (6.2)
3	6 (3.6)	5 (3)	1 (0.6)	2 (3.1)	0 (0)	2 (3.1)
Surgery_Met						
No	144 (85.2)	72 (42.6)	72 (42.6)	55 (84.6)	25 (38.5)	30 (46.2)
Yes	25 (14.8)	9 (5.3)	16 (9.5)	10 (15.4)	7 (10.8)	3 (4.6)

*TCC* transitional cell carcinoma, *PUC* papillary urothelial carcinoma, *SCC* small cell carcinoma

### Feature selection via LASSO

**Fig. 2:**
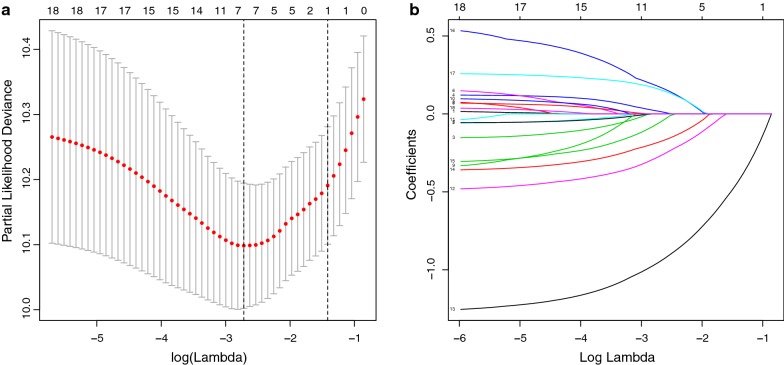
Clinical trait selection via the least absolute shrinkage and selection operator (LASSO) cox regression model. **a** Tenfold cross-validated error (first vertical line equals the minimum error (lambda = 0.066), whereas the second vertical line shows the cross-validated error within 1 standard error of the minimum). **b** The profile of coefficients in the model at varying levels of penalization plotted against the log (lambda) sequence

LASSO with tenfold cross-validation generated 7 variables out of 18 features: Grade, Surgery_Primary, Chemotherapy, Radiation_Therapy, Paliative_Care, Brain_Confined_Met, and CDCC_Score (Fig. 2a, b). The results of the univariate and multivariate Cox regression of the primary cohort are recorded in Table 3. Surgery_Primary, Chemotherapy, Radiation_Therapy, Paliative_Care, Brain_Confined_Met, and CDCC_Score were chosen for further analysis (apart from Radiation_Therapy, all other variables were independent prognostic factors in the LASSO Cox model; the reason for including this variable will be explained in “Discussion”). P values for Schoenfeld residuals method were all > 0.05 which fitted the proportional hazards assumption for the Cox model (Additional file 1: Fig. S1).

**Table 3:** Univariate and multivariate Cox regression analysis of BCa patients based on clinicopathological characteristics derived from NCDB data in the training cohort

Characteristics	Univariate analysis HR (95% CI)	P value	Multivariate analysis HR (95% CI)	P value
Age (< 65 years vs. ≥ 65 years)	1.117 (0.819–1.525)	0.48	1.032 (0.705–1.511)	0.87
Sex (male vs. female)	0.861 (0.602–1.233)	0.42	1.166 (0.756–1.797)	0.49
Race				
White vs. black	0.871 (0.509–1.489)	0.61	0.956 (0.523–1.747)	0.88
White vs. others	0.889 (0.363–2.174)	0.80	0.524 (0.197–1.39)	0.19
Grade				
Well differentiated vs. poorly differentiated	0.896 (0.511–1.574)	0.70	1.317 (0.653–2.656)	0.44
Well differentiated vs. unknown	1.144 (0.636–2.057)	0.65	1.634 (0.753–3.546)	0.21
Histology				
TCC vs. PUC	0.851 (0.588–1.232)	0.39	1.181 (0.738–1.89)	0.49
TCC vs. SCC	1.083 (0.563–2.087)	0.81	1.495 (0.714–3.13)	0.29
TCC vs. others	0.916 (0.578–1.45)	0.71	0.629 (0.363–1.09)	0.10
Lymph_nodes				
No vs. yes	0.835 (0.564–1.234)	0.37	0.808 (0.51–1.28)	0.36
No vs. unknown	0.985 (0.682–1.422)	0.93	0.761 (0.485–1.196)	0.24
Lymph_Vas_Invasion				
No vs. yes	1.098 (0.658–1.832)	0.72	1.494 (0.816–2.736)	0.19
No vs. unknown	1.291 (0.859–1.94)	0.22	1.269 (0.764–2.107)	0.36
Tumor_Stage (low vs. high)	1.247 (0.704–2.21)	0.45	1.089 (0.536–2.211)	0.81
Met_Bone (no vs. yes)	1.026 (0.742–1.42)	0.88	0.61 (0.374–0.997)	0.05
Met_Liver (no vs. yes)	0.978 (0.683–1.4)	0.90	1.223 (0.761–1.966)	0.41
Met_Lung (no vs. yes)	1.317 (0.962–1.802)	0.09	0.878 (0.525–1.469)	0.62
Surgery primary				
Minimal invasive surgery vs. no surgery	1.44 (1.031–2.011)	0.03	2.529 (1.609–3.975)	< 0.001
Minimal invasive surgery vs. non-minimal invasive	0.923 (0.558–1.525)	0.75	1.253 (0.672–2.334)	0.48
Chemotherapy (no vs. yes)	0.353 (0.25–0.498)	< 0.001	0.213 (0.137–0.332)	< 0.001
Radiation_Therapy (no vs. yes)	0.723 (0.53–0.986)	0.04	0.708 (0.486–1.031)	0.07
Palliative_Care (no vs. yes)	0.922 (0.651–1.305)	0.65	0.631 (0.413–0.964)	0.03
Brain_Confined_Met (non–brain confined vs. brain confined)	1.248 (0.911–1.71)	0.17	2.229 (1.144–4.345)	0.02
CDCC_Score				
0 vs. 1	1.29 (0.886–1.878)	0.18	1.439 (0.929–2.23)	0.10
0 vs. 2	1.529 (0.798–2.926)	0.20	1.865 (0.861–4.038)	0.11
0 vs. 3	2.14 (0.932–4.91)	0.07	2.545 (1.035–6.256)	0.04
Surgery_Met (yes vs. no)	0.9 (0.58–1.396)	0.64	0.918 (0.546–1.542)	0.75

### Development and validation of the prognostic nomogram

**Fig. 3:**
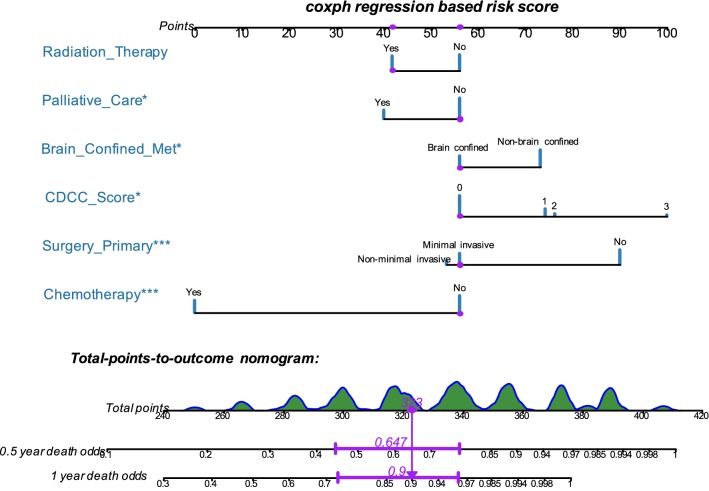
Nomogram to estimate the risk and predict the survival of BCa patients with brain metastasis. Bars in blue display the distribution of patients in the training cohort. To calculate the total points of a specific patient, locate the value of each variable on the top point axis, add the points from all of the variables, and draw a vertical line from the total point axis to determine the 0.5 and 1 year death probabilities at the lower line of the nomogram. Purple track provided an example for the calculation of total-points-to-outcome (*P < 0.05, **P < 0.01, ***P < 0.001)

**Fig. 4:**
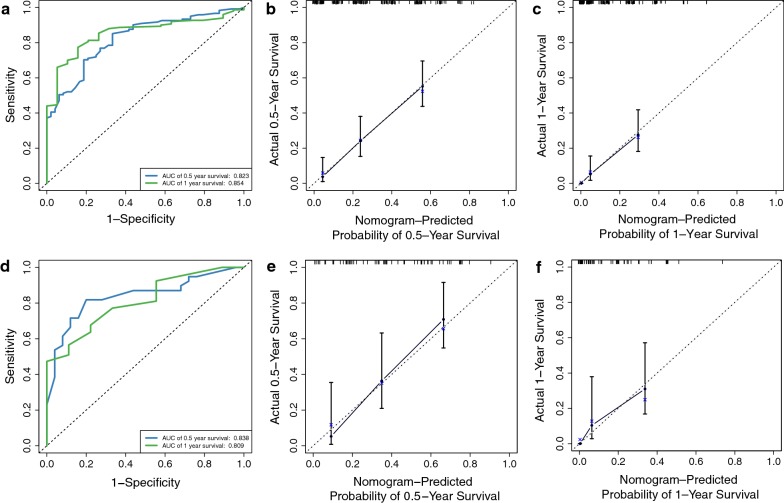
Time-dependent ROC curves comparing the prognostic accuracy of nomogram in BCa patients with metastatic brain lesions in the training cohort (**a**) and validation set (**d**). Validity of the predictive performance of the nomogram in the training cohort (**b**, **c**) and validation set (**e**, **f**). Nomogram-predicted probability of overall survival is plotted on the *x*-axis; actual overall survival is plotted on the *y*-axis. *ROC* receiver operator characteristic, *AUC* area under the curve

The prognostic nomogram that integrated all selected factors for OS in the primary cohort is shown in Fig. 3. We then derived a formula to calculate the risk score for odds of death for every patient based on their individual status of the selected variables above. To take one patient for example (purple track in Fig. 3), basing on the selected features, the total points adds up to 323 and thus the corresponding 0.5- and 1-year death probabilities are 0.647 and 0.9 respectively. The equation of each variable and computational formula is presented in Table 4. We further stratified those patients with an average or higher-than-average risk score into the high risk group and those with lower-than-average risk score into the low risk group (Table 1). In terms of 0.5- and 1-year OS of the training set, our six-clinical variable-based classifier demonstrated favorable discrimination with AUC values of 0.823 (95% confidence interval [CI] 0.758–0.889, P < 0.001) and 0.854 (95% CI 0.785–0.924, P < 0.001), respectively (Fig. 4a). The internal-bootstrapped calibration plot for the probability of survival at 0.5 or 1 year after surgery showed an optimal agreement between prediction by nomogram and actual observation (Fig. 4b, c). In the validation cohort, the nomogram displayed similar AUC values of 0.838 (95% CI 0.738–0.937, P < 0.001) and 0.809 (95% CI 0.680–0.939, P < 0.001) for the estimation of survival (Fig. 4d). There was also a well-behaved calibration curve for the survival estimation (Fig. 4e, f).

**Table 4:** The risk point of each variable and computational formula of OS

Clinical variables	Values	Risk points
Radiation_Therapy	No	56
	Yes	42
Palliative_Care	No	56
	Yes	40
Brain_Confined_Met	Non-brain confined	73
	Brain confined	56
CDCC_Score	1	56
	2	74
	3	76
	4	100
Surgery_Primary	No	90
	Minimal invasive	56
	Non-minimal invasive	53
Chemotherapy	No	56
	Yes	0

0.5-Year Survival = 7.5e−08 * points ^3 − 2.7837e−05 * points ^2 − 0.001082565 * points + 0.815518912

1-Year survival = 1.21e−07 * points ^3 − 2.3544e−05 * points ^2 − 0.003130703 * points + 0.651899934

**Fig. 5:**
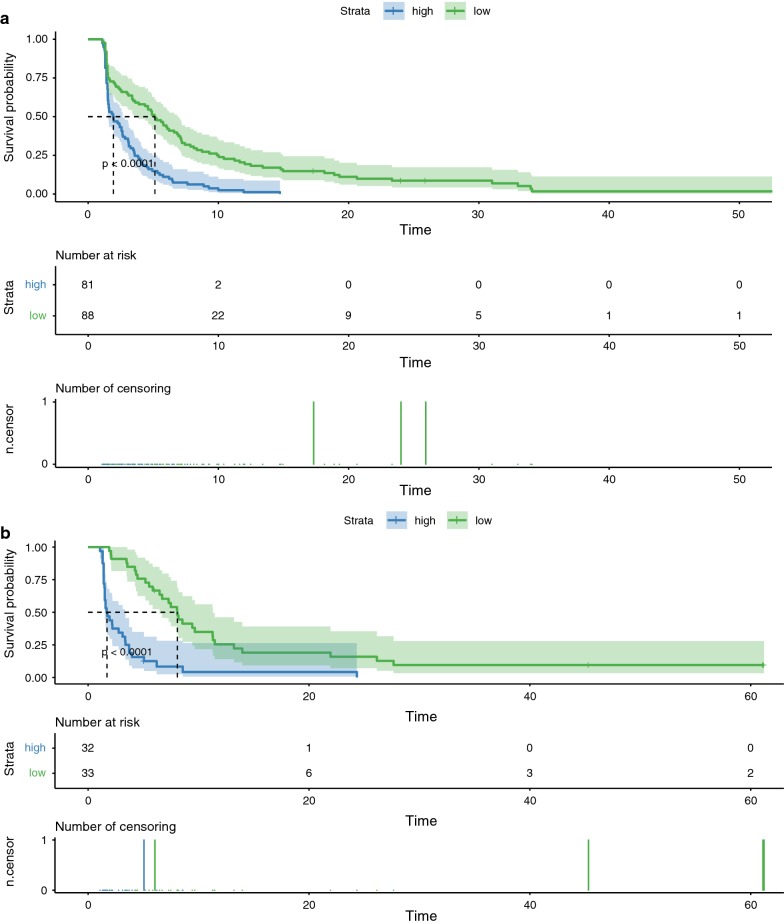
Kaplan–Meier survival analysis for all patients according to our classifier stratified by clinicopathological risk factors. Survival curves show the overall survival of high risk (blue) and low risk (green) groups between the training cohort (**a**) and validation set (**b**). Confidence interval band and risk table are also added

Kaplan–Meier survival analysis yielded a significant difference in survival between the training cohort and validation set. The median survival of the High and Low risk groups were 1.91 and 5.09 months in the training cohort (Fig. 5a) and 1.68 and 8.05 months in the validation set (Fig. 5b), respectively (both P < 0.0001).

**Fig. 6:**
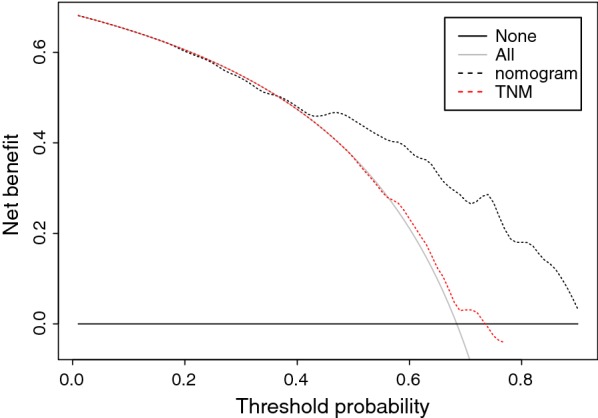
Decision curve analysis for OS. Black line: all victims dead. Gray line: none victims dead. Black dashed line: model of the nomogram. Red dashed line: staging system of TNM

Moreover, the decision curve analysis demonstrated that when the threshold probability was greater than 0.4, the nomogram presented more net benefit than TNM system in terms of OS prediction (Fig. 6).

## Discussion

In this study, we developed and validated a novel prognostic tool based on six clinical variables to improve the prediction of OS for patients with confirmed BCa with metastatic brain lesions. Our results showed that this tool can well categorize patients into high-risk and low-risk groups with large differences in OS.

Generally, in our research, prognostic factors are closely related to the choices of treatment modalities, as well as the comorbidities and metastatic conditions of the patient. Known as the best method of determining comorbidity conditions, higher Charlson/Deyo Score (CDCC_Score) is reported as a poor prognostic factor for overall mortality and cancer-specific mortality in metastatic BCa [15], consistent with our findings. In a previous study, multisite metastasis was found to be able to independently predict worse OS compared with single metastatic sites in BCa patients [5]. Our results are in line with the study above since brain-confined metastatic disease was related with better survival.

Treatment for the metastatic group is not beyond dispute. Our study included 4 treatment variables: Surgery_Primary, Surgery_Met, Chemotherapy, Paliative_Care and Radiation_Therapy.

A previous study indicated that surgical management of the primary BCa might contribute to long-term disease-free survival in selected patients [16]. Chen et al. also suggested that surgical management of the primary BCa might improve OS outcomes among patients [17]. Our study uncovered that brain metastatic BCa patients can still benefit from surgical operation of the primary site via minimal invasive surgery or otherwise. As for surgeries of the metastatic site, limited conclusions could be drawn for the lack of unanimous reporting elements and resection of solitary lung metastasis may result in OS improvement when integrated with chemotherapy [18]. As shown in the nomogram, though resistance may easily show up, chemotherapy still exerts maximal survival benefit for brain-metastatic BCa patients, which correlates to the first-line treatment of the European Association of Urology guidelines [19]. Given that more than half of patients with metastatic urothelial cancer are unfit for cisplatin-based chemotherapy, the choice of chemotherapy combination will have to depend on the health condition of patients. Consensus from an international survey among urologic experts was reached to define patients unfit for cisplatin-based chemotherapy, which was as follows: performance score > 1, glomerular filtration rate ≤ 60 ml/min/1.73 m^2^, grade 2 audiometric loss and peripheral neuropathy, and New York Heart Association class III heart failure [20, 21]. Palliative care is defined as any procedures to alleviate symptoms distinguishable from the same modality used for curative intent, which may include surgery, radiation therapy, systemic therapy, and/or other pain management drugs. Advanced BCa can be associated with problems like ureteral obstruction, persistent bleeding, pain, and/or voiding complaints; palliative care may prolong life expectancy in these patients [22]. The variable Radiation_Therapy was fitted into the analysis because although the P value 0.07 slightly surpassed 0.05 in the multivariate Cox model, it was 0.04 in the univariate analysis. Moreover, for brain metastatic cancer, conventional fractionated whole brain radiotherapy is still frequently used as a standard therapy [23]; thus, we included it in the prognostic nomogram for clinical consideration.

To the best of our knowledge, this is the largest cohort study exploring the prognostic significance of BCa with brain metastasis and the effect of various treatments on patients’ prognoses; however, several limitations are still noteworthy. For example, information regarding metastasectomy for specific metastatic sites was incomplete. In addition, there was a lack of details and sequences concerning chemotherapy, endocrine therapy, immunological treatment, and radiation therapy. As a retrospective study population from different medical facilities, some baseline characteristics may be non-uniform and external validation cohorts are needed to confirm the predictive accuracy of the nomogram.

## Conclusion

By combining six clinical factors of brain-metastatic BCa patients, we constructed a prognostic nomogram. The model provides an optimal estimation of OS and reference for suitable treatments in BCa patients with brain metastasis.

## Supplementary information

**Additional file 1: Fig. S1.** The graphical verification of proportional hazards assumption for the Cox regression model.

## Data Availability

Original data for BCa patients with brain metastasis can be found at http://dx.doi.org/10.17632/nn6y58v8vv.1#file-a72735e9-15b5-4a10-aef5-deddad2463e8.

## Abbreviations

BCa: bladder cancer
AUC: area under the receiver operating characteristic curve
CRP: C-reactive protein
OS: overall survival
NCDB: National Cancer Database
LASSO: least absolute shrinkage and selection operator
CI: confidence interval
CDCC_Score: Charlson/Deyo Score

## References

[1] Siegel RL, Miller KD, Jemal A (2017). Cancer statistics, 2017. CA Cancer J Clin.

[2] Stein JP (2001). Radical cystectomy in the treatment of invasive bladder cancer: long-term results in 1,054 patients. J Clin Oncol Off J Am Soc Clin Oncol.

[3] von der Maase H (2005). Long-term survival results of a randomized trial comparing gemcitabine plus cisplatin, with methotrexate, vinblastine, doxorubicin, plus cisplatin in patients with bladder cancer. J Clin Oncol Off J Am Soc Clin Oncol.

[4] Sternberg CN, Vogelzang NJ (2003). Gemcitabine, paclitaxel, pemetrexed and other newer agents in urothelial and kidney cancers. Crit Rev Oncol Hematol.

[5] Dong F (2017). Prognostic value of site-specific metastases and therapeutic roles of surgery for patients with metastatic bladder cancer: a population-based study. Cancer Manag Res.

[6] Tsao MN (2012). Radiotherapeutic and surgical management for newly diagnosed brain metastasis(es): an American Society for Radiation Oncology evidence-based guideline. Pract Radiat Oncol.

[7] Rades D (2008). Comparison of stereotactic radiosurgery (SRS) alone and whole brain radiotherapy (WBRT) plus a stereotactic boost (WBRT + SRS) for one to three brain metastases. Strahlenther Onkol Organ Dtsch Rontgengesellschaft Al.

[8] Brown PD (2016). Effect of radiosurgery alone vs radiosurgery with whole brain radiation therapy on cognitive function in patients with 1 to 3 brain metastases: a randomized clinical trial. JAMA.

[9] Mahmoud-Ahmed AS (2002). Brain metastases from bladder carcinoma: presentation, treatment and survival. J Urol.

[10] Fokas E, Henzel M, Engenhart-Cabillic R (2010). A comparison of radiotherapy with radiotherapy plus surgery for brain metastases from urinary bladder cancer: analysis of 62 patients. Strahlenther Onkol Organ Dtsch Rontgengesellschaft Al.

[11] Bellmunt J (2010). Prognostic factors in patients with advanced transitional cell carcinoma of the urothelial tract experiencing treatment failure with platinum-containing regimens. J Clin Oncol Off J Am Soc Clin Oncol.

[12] Ishioka J (2012). Development of a nomogram incorporating serum C-reactive protein level to predict overall survival of patients with advanced urothelial carcinoma and its evaluation by decision curve analysis. Br J Cancer.

[13] Balachandran VP, Gonen M, Smith JJ, DeMatteo RP (2015). Nomograms in oncology: more than meets the eye. Lancet Oncol.

[14] Tibshirani R (1996). Regression shrinkage and selection via the Lasso. J R Stat Soc Ser B Methodol.

[15] Mayr R (2012). Predictive capacity of four comorbidity indices estimating perioperative mortality after radical cystectomy for urothelial carcinoma of the bladder. BJU Int.

[16] Herr HW, Donat SM, Bajorin DF (2001). Post-chemotherapy surgery in patients with unresectable or regionally metastatic bladder cancer. J Urol.

[17] Chen C, Hu L, Chen Y, Hou J (2017). The prognostic value of histological subtype in patients with metastatic bladder cancer. Oncotarget.

[18] Nakagawa T (2017). Oncologic outcome of metastasectomy for urothelial carcinoma: who is the best candidate?. Ann Surg Oncol.

[19] Alfred Witjes J (2017). Updated 2016 EAU guidelines on muscle-invasive and metastatic bladder cancer. Eur Urol.

[20] Hussain SA (2012). A study of split-dose cisplatin-based neo-adjuvant chemotherapy in muscle-invasive bladder cancer. Oncol Lett.

[21] Morales-Barrera R (2012). Cisplatin and gemcitabine administered every two weeks in patients with locally advanced or metastatic urothelial carcinoma and impaired renal function. Eur J Cancer Oxf Engl.

[22] Ghahestani SM, Shakhssalim N (2009). Palliative treatment of intractable hematuria in context of advanced bladder cancer: a systematic review. Urol J.

[23] Tsao MN (2012). International practice survey on the management of brain metastases: third international consensus workshop on palliative radiotherapy and symptom control. Clin Oncol.

