# Supplementary figures and images for: Prognostic nomogram for bladder cancer with brain metastases: a National Cancer Database analysis
Source: J Transl Med. 2019 Dec 9;17:411. doi: 10.1186/s12967-019-2109-7 (PMC6902467; doi:10.1186/s12967-019-2109-7)

# Training Cohort: Global Schoenfeld Test p: 0.91

**A**

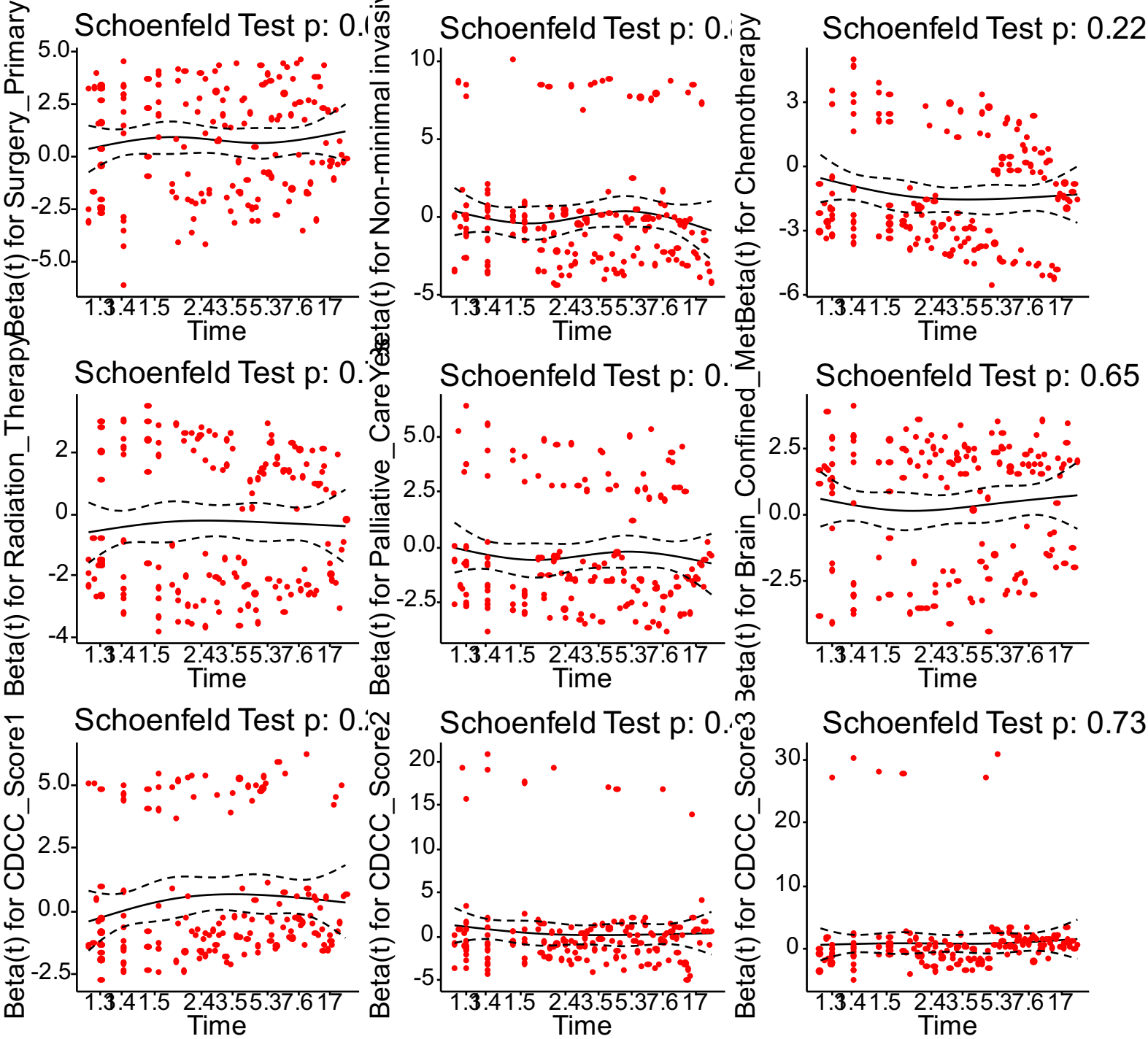

**B**

## Testing Cohort: Global Schoenfeld Test p: 0.53

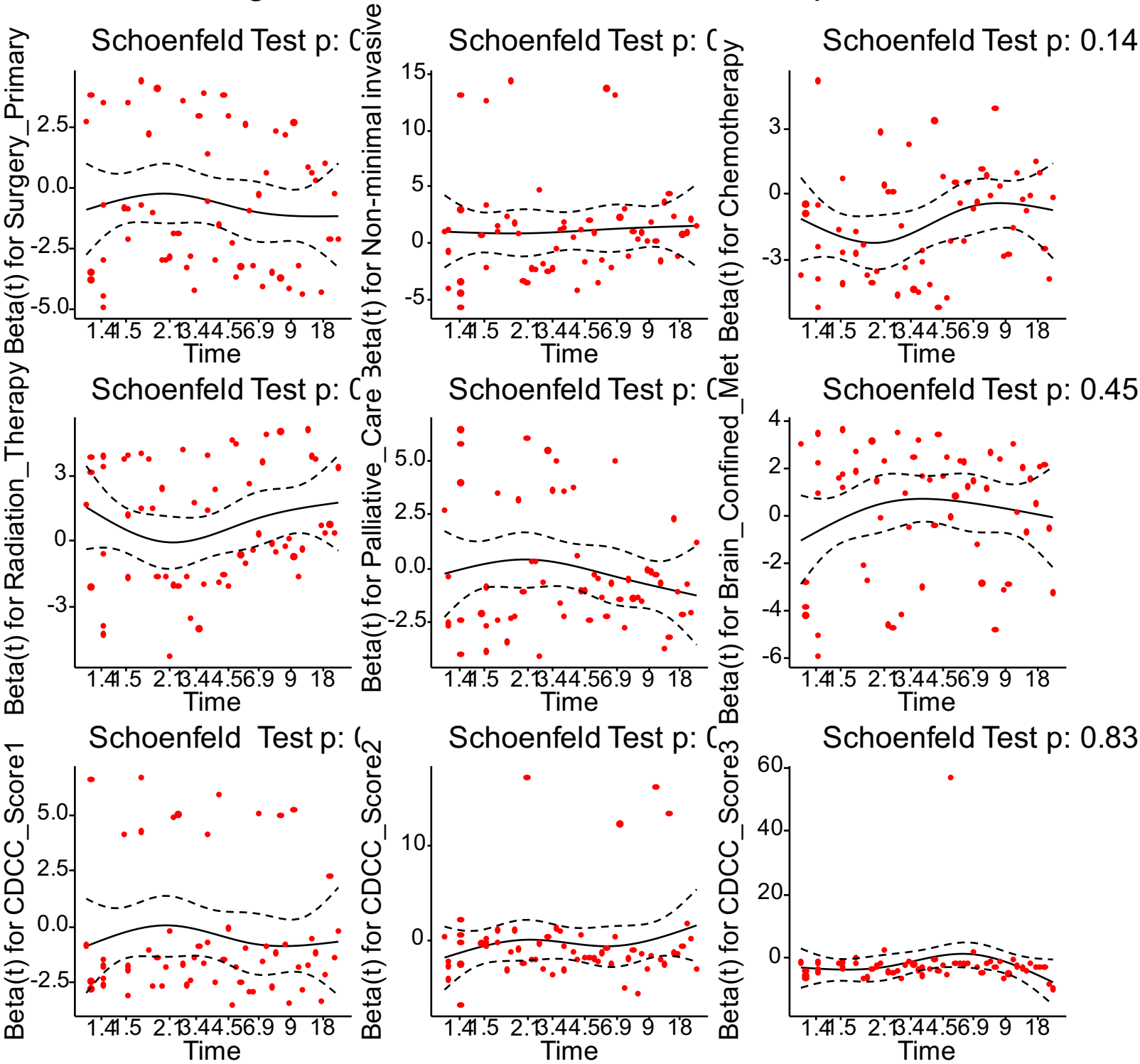

Supplement: Supplementary file 1 — Additional file 1: Fig. S1. The graphical verification of proportional hazards assumption for the Cox regression model. [file 12967_2019_2109_MOESM1_ESM.pdf]
